# Efficiency of Biological Typing Methods in Maize Hybrid Genetic Purity Estimation

**DOI:** 10.3390/genes14061195

**Published:** 2023-05-30

**Authors:** Anika Kovincic, Ksenija Markovic, Danijela Ristic, Vojka Babic, Tanja Petrovic, Tomislav Zivanovic, Natalija Kravic

**Affiliations:** 1 Maize Research Institute Zemun Polje, Slobodana Bajica 1, 11185 Belgrade, Serbia; anisavic@mrizp.rs (A.K.); kmarkovic@mrizp.rs (K.M.); dristic@mrizp.rs (D.R.); vbabic@mrizp.rs (V.B.); ptanja@mrizp.rs (T.P.); 2 Faculty of Agriculture, University of Belgrade, Nemanjina 6, 11080 Belgrade, Serbia; tomislav@agrif.bg.ac.rs

**Keywords:** phenotypic uniformity, seed storage proteins, SSRs, ultrathin-layer isoelectric focusing, *Zea mays* L.

## Abstract

A high level of genetic purity in crop varieties must be achieved and maintained for agronomic performance, encouraging investment and innovation in plant breeding and ensuring that the improvements in productivity and quality imparted by breeders are delivered to the consumer. Since the success of hybrid seed production is dependent upon the genetic purity of the parental lines, in this study, the experimental F1*exp* maize hybrid and its parental inbreeds were used as a model system to examine the discriminative power of morphological, biochemical and SSR markers for seed purity assay. The highest number of off-type plants was estimated by morphological markers. According to the comparison of prolamins and albumins banding patterns of parental and derived F1*exp* seeds, genetic impurities could not be detected. Molecular analysis detected two types of genetic profile irregularity. Beside its use for verifying varieties of maize, report on *umc*1545 primer pair ability to detect non-specific bands (i.e., off-types), in both the maternal component and F1*exp*, which is the first report on this issue yet, strongly supports the recommendation of this SSR marker use for more accurate and time-efficient maize hybrids and parental lines genetic pyrity testing.

## 1. Introduction

Maize (*Zea mays* L.) is the most important cereal crop in the world, used for food, fodder, medical purpose and as a biofuel. Hybrid variety is the first generation of crossing genetically distant parental components. To ensure maximum kernel set and high level of genetic purity during hybrid seed production, much effort has always been directed towards managing the process [1]. Previous cropping, isolation, field inspection, varietal identity and varietal purity are the basic requirements for maize hybrid production. Well-organized seed certification system is necessary, which includes field inspection of crops during the growth season, as well as laboratory seed testing, where both indicators require declaration as a basis for placing seeds on the market [2]. It is estimated that a drop in cultivated maize hybrid seed purity by less than 1% can result in a loss of 135 kg per hectare [3]. Parent-offspring test helps to prove parentage for a specific hybrid whether it is a true derivative of the original parental inbred lines without pollen contamination. The choice of a method / marker for the assessment of genetic purity depends on its ability to identify the genotype, repeatability, technical complexity, cost and time required to perform the experiment.

Different strategies for plant varietal and purity assessment are very important in breeding, registration and trade process [4,5,6]. Traditionally, morphological comparison has formed the basis for genetic purity evaluations. The UPOV (International Union for the Protection of New Varieties of Plants) descriptor for maize is one of the several guidelines for standardization of maize morphological description [4]. Morphological markers are time-consuming, highly dependent on environmental factors and are susceptible to phenotypic plasticity. Despite all that, morphological traits are still very important in determination of the agronomic value and in taxonomic classification of plant species, including maize [7]. Ultrathin-layer isoelectric focusing (UTLIEF) is a standard reference method for testing the genetic purity of lines and hybrids [8]. This method consists of extracting seed storage proteins (water soluble—albumins and alcohol soluble—prolamins) from individual kernels and their separation on a pH gradient polyacrylamide gel, according to differences in isoelectric point. ISTA (International Seed Testing Association) accepted this technique as the standard technique for varietal identification and genetic purity testing of maize and sunflower [9]. Molecular markers, as highly precise, time- and resource-saving technology, not stage- or tissue-specific and not affected by the environment, have been used in crop breeding and genetic purity testing [5]. The simple sequence repeats (SSRs) are co-dominant, highly polymorphic and very informative markers, and thus, of great importance for the rapid assessment of seed purity of hybrid and parental lines [10].

Since the best way to maintain genetic purity in maize hybrid seed production is to maintain high genetic purity level of its parental components, the aim of this study is: (i) to evaluate the varietal purity of one experimental maize hybrid and its parental lines using three types of biological markers (morphological and biochemical markers, as well as SSR markers prescribed by ISTA for maize variety verification) and (ii) to compare marker types’ efficiency regarding genetic purity assessment.

## 2. Materials and Methods

### 2.1. Plant Material

In 2019, genetic purity of one experimental maize hybrid (F1*exp*) was tested by UTLIEF method. In parallel, the phenotypic uniformity of the same hybrid was evaluated by morphological markers according to UPOV descriptor. The hybrid expressed uniform protein marker profiles, while unsatisfactory uniformity of morphological markers.

The next year (2020), for more accurate evaluation of phenotypic uniformity and genetic purity, the same F1*exp* hybrid (i.e., the same seed lot used for 2019 sowing season) along with its parental components were subjected to field trial and laboratory testing.

### 2.2. Varietal Uniformity Estimation by Morphological Markers

The open-field experiment was conducted at Zemun Polje, Serbia (44°52′00″ latitude N, 20°19′00″ longitude E, 81 m altitude) in 2020, on Calcaric Chernozem of silty loam texture. The experiment was set up according to randomized complete block design, in two replications. Each genotype was sown in 2 rows, with 20 plants per row. Intra-row and inter-row separation was 0.20 m and 0.75 m, respectively. The uniformity of each genotype was assessed by the observation of all relevant morphological traits (Table 1) according to the UPOV descriptor [11], on 20 randomly chosen individual plants per replication (i.e., in total 40 plants per trial). According to method of varietal propagation and type of morphological traits expression method, the off-types and the standard deviations (STDEV) methods [12] were used for varietal uniformity assessment. In this study, the STDEV approach was applied for metrically measured (MS) traits, while the off-types approach was used for the visually assessed (VS) traits (Table 1). Moreover, for inbred lines and single-cross hybrid uniformity assessment, a population standard of 3% and an acceptance probability of at least 95% was applied [11]. Namely, for samples of 40 plants in size (as was the case in this experiment), 3 off-type plants are allowed [13].

The “population standard” can be expressed as the maximum percentage of off-types to be accepted if all individuals of the variety could be examined. The “acceptance probability” is the minimum probability of accepting as uniform a variety with the population standard of off-types. Irrespective of the number of morphological traits for which a plant has an obviously different expression from original varietal type, it will only be counted as one off-type plant. According to the STDEV approach for varietal uniformity assessment, a candidate variety should not be significantly less uniform than the comparable ones as the varieties of the same type within the same or a closely related species that has been previously examined and considered to be uniform [12]. In this study, the comparison between a candidate and comparable varieties was performed on the STDEV basis, calculated for MS traits of individual plant observations. When making a decision on uniformity level based on relative standard deviations (i.e., a ration between standard deviation of candidate vs. comparable variety), defined threshold value for sample of 40 plants in size is 1.26 [13]. For the comparable varieties, single-cross hybrid (F1*c*) and commercial inbred line (L*c*), estimated as uniform in the Maize Research Institute Zemun Polje (MRIZP) field trials for seed production control, were used.

### 2.3. Genetic Purity Estimation by Protein Markers

For genetic purity testing using UTLIEF, 400 kernels of F1*exp* and 100 kernels of each parental line were analyzed, as described in chapter 8.9.3 of ISTA rules [14]. For estimation of maize hybrid genetic purity, marker protein bands differing among the hybrid parental lines must be analyzed. Determination of informative fraction of seed storage proteins (albumins or prolamins) is also required for acquiring more accurate band legibility. A legible reference gel enables detection of protein bands in male parent lacking in female parent, thus enabling reliable estimation of hybrid genetic purity. In this study, the albumin and prolamine electrophoregrams containing proteins isolated from 32 seeds of each male and female parent, together with proteins isolated from 32 F1*exp* hybrid seeds, were considered as the reference gels.

Ultrathin (0.12 mm) gradient polyacrylamide gels were cast on polyester support films (Gel-Fix, Serva). The polymerization solution for 10 gels contained 16 g urea, 50 ml acrylamide (T = 6.8%, C = 2.5%), 4.4 ml of pH 5–8/2–11 ampholytes (Servalyt 5-8 and Servalyt 2-11, Serva), 50 µl N NN’ N’-tetramethylethylenediamine and 350 µl of 20% (*w/v*) ammonium persulphate. Electrophoresis was carried out on a horizontal electrophoresis unit (Multiphor II, GE Helathcare, Chicago, IL, USA) connected to a cooling apparatus (MultiTemp III, GE Healthcare) at 6.5 °C. Each gel was divided into two focusing zones using one cathode in the middle and two anodal electrodes the sides of the gel. The anode solution contained 0.332% (*w/v*) aspartic acid and 0.368% (*w/v*) glutamic acid, and the cathode solution 0.472% (*w/v*) arginine (base), 0.364% (*w/v*) lysine and 12% (*v/v*) ethylenediamine. The application strips (52 wells) were positioned 0.5 cm apart from the anodes. In amount of 20 µl of each samples’ supernatant were pipetted into the applicator wells. Electrophoresis (focusing) was run at 2500 V, 15 mA, 40 W for ~1750 volt/hours. After focusing, gels were fixed in 12% (*w/v*) trichloroacetic acid for 20 min, then stained in Coomassie Brilliant Blue solution containing 0.015% (*w/v*) Coomassie R 250 and 0.045% (*w/v*) Coomassie G 250, 11% (*v/v*) acetic acid, 18% (*v/v*) ethanol and 71% (*v/v*) water for 50 min, and finally, destained in a solution of 30% (*v/v*) ethanol, 5% (*v/v*) acetic acid and 65% (*v/v*) water for 10 min. The gels were air dried overnight at room temperature.

### 2.4. Genetic Purity Estimation by Molecular Markers

The same plants evaluated by morphological markers (i.e., 40 plants per genotype) were used for SSRs analysis. Genomic DNA was isolated from fresh leaf tissue of individual plants using commercial NuceoSpin Plant II Macherey-Nagel kit. A total of 10 informative SSR markers were chosen for the analysis, out of which 8 are prescribed by the ISTA [15] for verifying varieties of maize (Table 2). PCR amplification reaction was carried out in 25 µl reaction volume containing DreamTaq™ Green PCR Master Mix (2X) (Thermo Scientific, Waltham, MA, USA), 0.5 µM primers and 1µL of DNA. Amplification was conducted using the “Touchdown” program as follows: an initial denaturation at 94 °C/10 min, followed by 10 cycles each of denaturation at 94 °C/30 s, annealing at 64 °C/1 min (−1 °C/cycle) and extension at 72 °C/30 s; another 30 cycles of 94 °C/30 s, 55 °C/30 s and 72 °C/30 s were performed. Final extension was performed at 72 °C for 10 min. Amplified fragments were separated on 8% polyacrylamide gels with 20 bp DNA marker ladder (Thermo Scientific) on a vertical gel system (Mini Protean Tetra-Cell BioRad, Hercules, CA, USA) and stained with ethidium bromide. The gels were photographed using BioDocAnalyze (BDA) gel documentation system (Biometra, Göttingen, Germany) and SSR profiles for each primer were determined.

## 3. Results

### 3.1. Varietal Uniformity Estimation by Morphological Markers

For uniformity assessment of nine quantitative (MS) traits, the STDEV approach was applied, considering comparison of the candidate varieties (i.e., F1*exp*, F1♀ and F1♂) with the corresponding comparable ones (i.e., F1*c* and L*c*) (Table 3).

Although the STDEV values for the width of blade (WB), length of ear (LE) and the number of rows of grain (NR) in the experimental hybrid (F1*exp*) and for the number of primary tassel branches (NPTB) in the maternal component exceeded the STDEV value for the same traits in the comparable varieties (i.e., F1*c* and L*c*, respectively), they did not exceed the defined threshold value of 1.26 for a sample of 40 plants in size (as was the case in this study). On the other hand, STDEV values for all evaluated traits achieved by the paternal inbred were below the STDEV values expressed by the comparable line (L*c*).

Visual assessment of uniformity encompassed the counting of off-types for each trait, while combining two replications (i.e., 40 plants per genotype, i.e., per trial). The results of phenotypic uniformity based on visual assessments of 22 examined traits are presented in Table 4.

The F1*exp* plants expressed variability in four traits: the anthocyanin coloration of silks (ACS), the anthocyanin coloration of brace roots (ACBR), the type of grain (TG) and the color of top of grain (CTG). A slight variation in these traits, which was not considered as an off-type variation, was the following: variation in ACS, observed in 15 plants rated 5 (medium), i.e., 20 plants rated 3 (weak); in ACBR, observed in 31 plants rated 5 (medium), i.e., 8 plants rated 7 (strong); in TG, observed in 13 plants rated 4 (dent-like) i.e. 22 plants rated 5 (dent), as well as in CTG, observed in 26 plants rated 3 (yellow), i.e., 14 plants rated 4 (yellow orange), respectively. However, highly pronounced variation, which must certainly be considered as an off-type variation, was the following: variation in anthocyanin coloration of tassel anthers (ACA), recorded in three plants rated one (absent or very weak), i.e., one plant rated seven (strong); in ACS, also recorded in three plants rated one (absent or very weak), i.e., one plant rated seven (strong), as well as in TG, observed in five plants rated three (intermediate), respectively. In the case of ACBR, the length of lateral branch (LLB) and the dorsal side of grain (CDSG), the majority of plants (39 of them) exhibited the uniformity in trait expression. In total, 14 off-type plants regarding the evaluated traits were recorded in F1*exp* (Table 4).

The majority of F1♀ plants exhibited slight variation in the ACS and ACBR traits. A highly pronounced variation was recorded as two off-type plants rated 3 (weak) for ACBR trait. Off-type variation was also observed for the following traits: the anthocyanin coloration of tassel anthers (ACA), recorded in two plants rated one (absent or very weak); the density of tassel spikelets (DTS), noticed in one plant rated three (moderately lax) and the color of top of grain (CTG), registered in four plants rated four (yellow orange). In total, nine off-type plants were observed in F1♀ component (Table 4).

The F1♂ plants expressed a slight variation in the anthocyanin coloration of internodes (ACIn), observed in 17 plants rated 3 (weak), i.e., 23 plants rated 5 (medium). A highly pronounced variation was recorded as one off-type plant rated 3 (weak) for the anthocyanin coloration of tassel glume excluding base (ACG) (Table 4).

According to the results obtained, only paternal inbred line can be considered as uniform in traits’ expression on the basis of visual assessment.

### 3.2. Genetic Purity Estimation by Protein Markers

**Figure 1:**
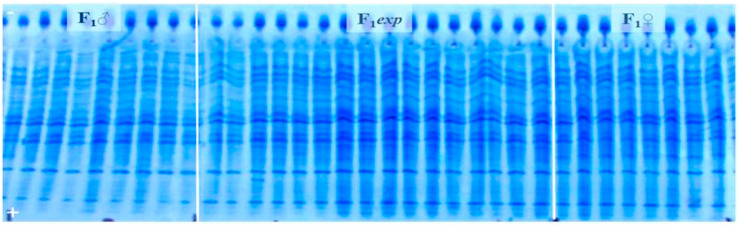
UTLIEF electrophoregram of water-extracted seed storage proteins (albumins) from experimental (F1*exp*) hybrid, female (F1♀) and (F1♂) male parent, (− anode, + cathode).

**Figure 2:**
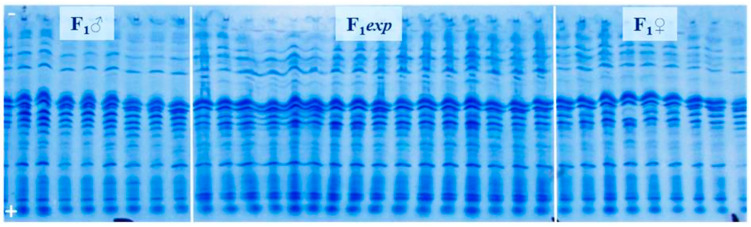
UTLIEF electrophoregram of alcohol-extracted seed storage proteins (prolamins) from experimental (F1*exp*) hybrid, female (F1♀) and (F1♂) male parent, (− anode, + cathode).

The electropherogram containing proteins isolated from 32 seeds of both male (F1♂) and female (F1♀) parent, together with proteins isolated from 32 (F1*exp*) hybrid seeds, was considered to be the reference gel. Figure 1 and Figure 2 are illustrations of albumin profiles and prolamine profiles, respectively. Through visualization and comparison of albumin and prolamin protein patterns upon UTLIEF electrophoresis (pH 5–8/2–11), it was found that the albumins could be more legible for genetic purity analysis of experimental F1*exp* hybrid. Moreover, informative male parent marker band in reference gels could not be detected following the visualization of either fraction of seed storage proteins.

**Figure 3:**
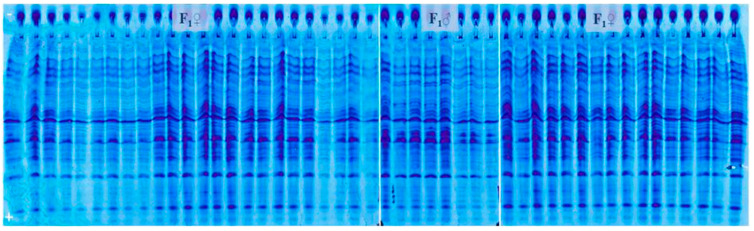
UTLIEF electrophoregram of parental components’ albumins (female—F1♀ and F1♂—male parent, − anode, + cathode).

Analyses of albumin banding patterns of maternal and paternal seeds (Figure 3) were done to enable the identification of male marker band, as well as potential heterozygosity presence within the parental inbred lines of F1*exp* hybrid. Neither specific male marker band nor heterozygosity of any of parental inbred line could be detected.

**Figure 4:**
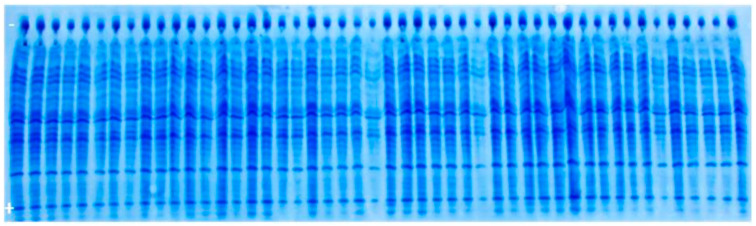
UTLIEF electrophoregram of experimental hybrid’s albumins (− anode, + cathode).

Based on the fact that male parent marker band could not be detected, we relayed on the possibility to find off-types corresponding to foreign pollination or contamination with another variety. For this purpose, 400 individual seeds’ albumins banding patterns of the experimental hybrid were analyzed (example Figure 4). As revealed by UTLIEF electrophoresis of all analyzed hybrid seeds, no differences within their albumin banding patterns could be revealed. Thus, it can be suggested that the tested experimental hybrid was genetically pure.

**Figure 5:**
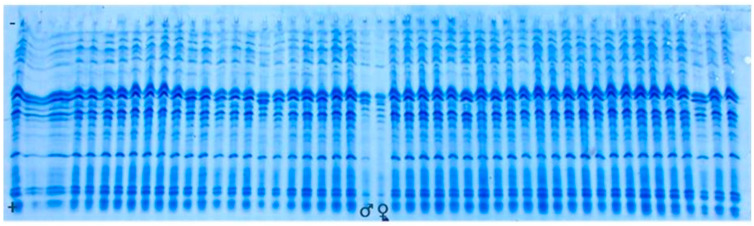
UTLIEF electrophoregram of experimental hybrid’s prolamins (F1♂ male parent, F1♀ female parent, − anode, + cathode).

To check the results, testing was repeated on prolamine fraction of 400 hybrid individual seeds, with parental components included on the gel (example Figure 5). According to the comparison of prolamins banding patterns of the parental lines and the tested experimental hybrid, genetic impurities could not be detected.

### 3.3. Genetic Purity Estimation by Molecular Markers

The genetic purity of experimental hybrid and its parental inbred lines was evaluated by eight SSR markers for variety verification in *Zea mays* prescribed by ISTA, along with two additional SSR markers previously found to be informative in genotyping at MRIZP. Three informative SSR markers in terms of detecting the polymorphism between the experimental hybrid and its corresponding parental lines are as follows: *umc*1133, *umc*1545 and *phi*015 (Table 5 and Table 6).

**Figure 6:**
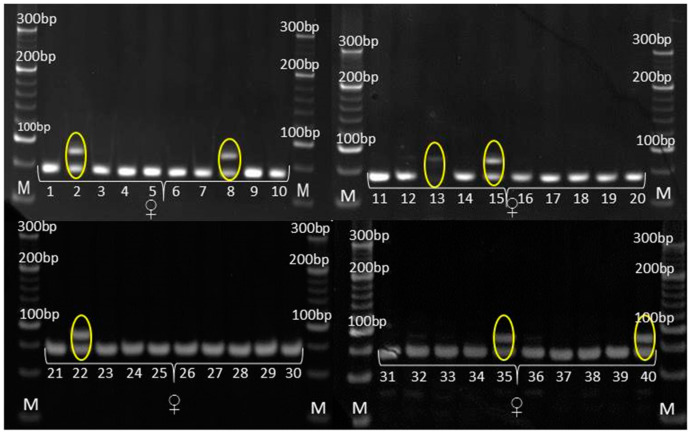
Amplification profile of primer pair *umc*1545 for F1♀ maize inbred line. M—DNA ladder (20 bp). The numbers of lanes 1 to 40 correspond to the F1♀ plant samples. Yellow framed off-type plant samples.

**Figure 7:**
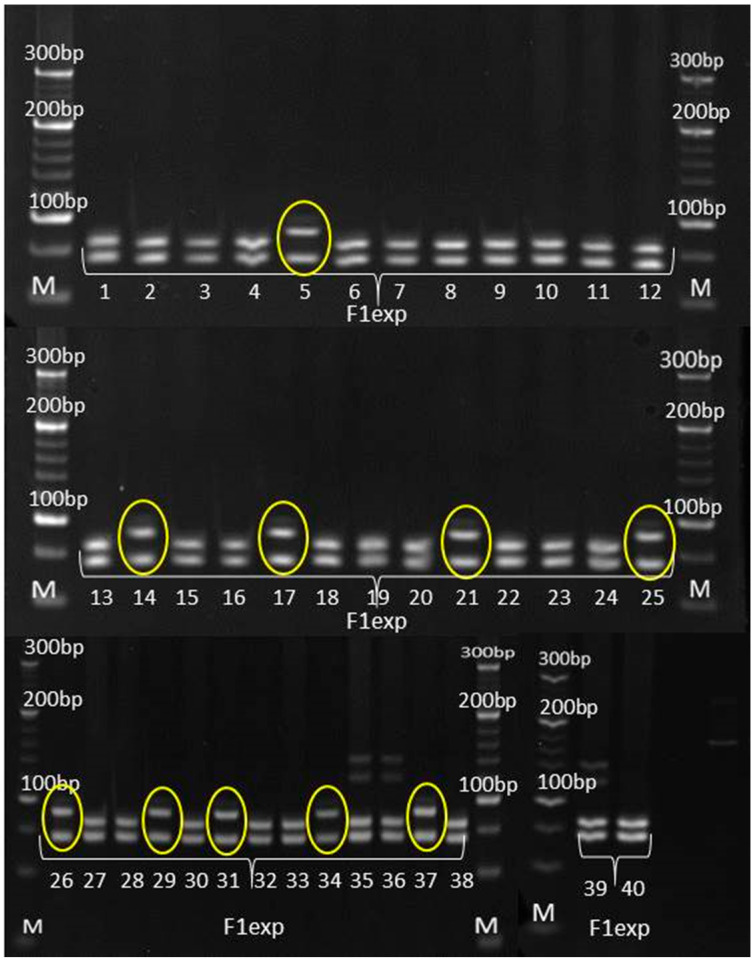
Amplification profile of primer pair *umc*1545 for F1*exp* maize hybrid. M—DNA ladder (20 bp). The numbers of lanes 1 to 40 correspond to the F1*exp* plant samples. Yellow framed off-type plant samples.

**Figure 8:**
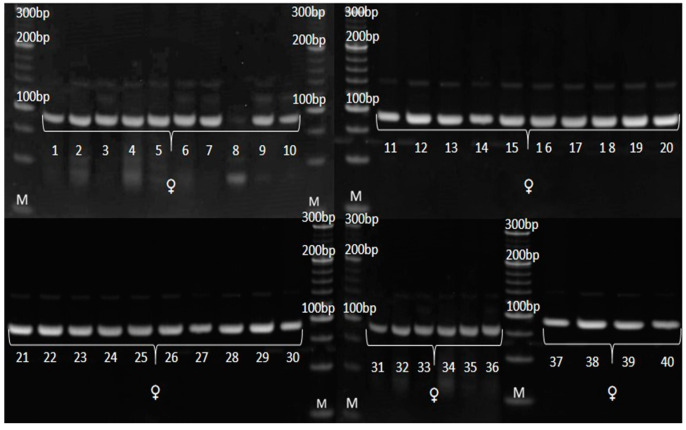
Amplification profile of primer pair *umc*1133 for F1♀ maize inbred line. M—DNA ladder (20 bp). The numbers of lanes 1 to 40 correspond to the F1♀ plant samples.

**Figure 9:**
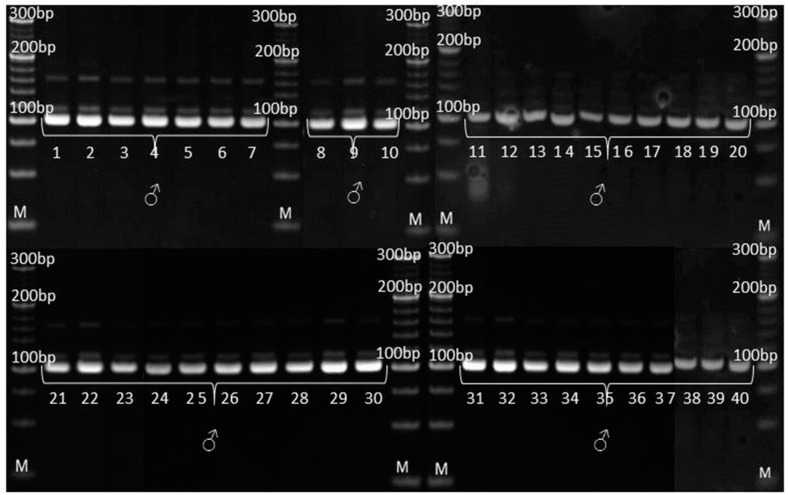
Amplification profile of primer pair *umc*1133 for F1♂ maize inbred line. M—DNA ladder (20 bp). The numbers of lanes 1 to 40 correspond to the F1♂ plant samples.

**Figure 10:**
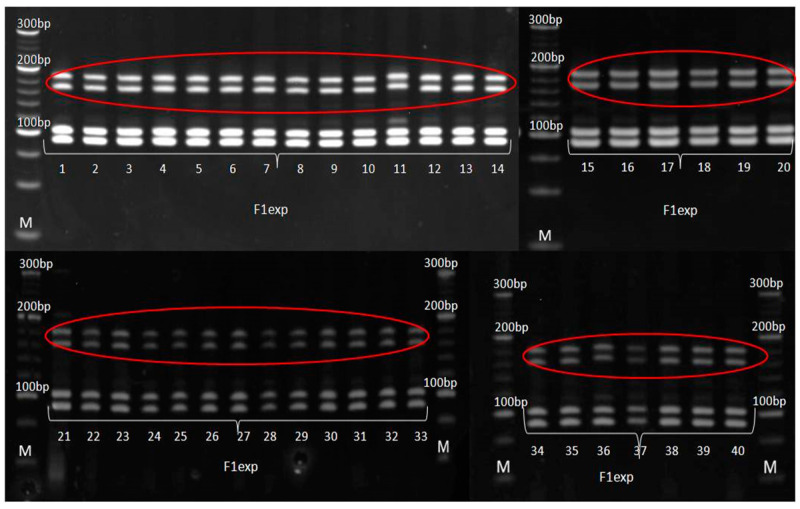
Amplification profile of primer pair *umc*1133 for F1*exp* maize hybrid. M—DNA ladder (20 bp). The numbers of lanes 1 to 40 correspond to the F1exp plant samples. Red framed non-parental alleles.

According to SSR analysis, paternal inbred line exhibited uniform genetic profile. Molecular analysis determined the occurrence of two types of irregularity: (i) SSR that detected the existence of non-specific bands in the maternal inbred (Figure 6), also detected the presence of these bands in the experimental hybrid (Figure 7) and (ii) SSRs that detected the uniformity of parental lines (Figure 8 and Figure 9), detected the presence of non-parental inheritance in the F1*exp* hybrid (Figure 10).

It was observed that the genetic purity of F1*exp* determined by SSR analysis was of 75%, which was 10% higher in comparison to genetic purity determined by the visually assessed traits (Table 4). The results obtained from the SSR and morphological analyses for F1*exp* hybrid plant samples were not consistent for the following individuals: 3, 11, 20 and 32, respectively. Namely, three F1*exp* individuals (i.e., plant samples 3, 20 and 32) assessed as off-types for TG trait together with one off-type plant (i.e., plant sample 11) for ACS trait, were genetically pure according to *umc*1545 marker (Figure 7). For the F1♀ maize inbred line, the same marker determined 82.5% of its genetic purity, while the Off-type approach for VS traits detected for 5% lower genetic uniformity of F1♀ plant samples. In this case, the plant samples 5 and 27, detected as off-types for ACBR trait, were found to be pure by *umc*1545 marker (Figure 6).

## 4. Discussion

A high level of genetic purity provides levels of performance that meet predicted expectations; moreover, in maize, as a hybrid crop, there is the additional necessity of maintaining purity of F_1_ seed so that high performance is carried through to the commercial product. Without a high standard of purity for varieties and inbred lines, genotypes will have the opportunity to drift [16].

### 4.1. Varietal Uniformity Estimation by Morphological Markers

Despite the fact that the genetic basis of most morphological traits still remains unknown and that the morphological traits provide, at best, an indirect way of assessing genetic purity, their observation in field grownouts continues to be the most widely used approach for describing varieties de novo, identifying varieties and monitoring genetic purity [17].

In this study, the application of the appropriate descriptors (i.e., 31 morphological traits), a measurement type (i.e., metrical measurement—scale level of measurement, and visual assessment—ordinal level of measurement) and the biometric method (i.e., the STDEV and off-types approaches) resulted in higher quality information from morphological markers observed, which was in line with reported phenotypic characterization of maize inbred lines using UPOV descriptors [18].

According to the STDEV approach suitable for the determination of off-types using measurement of single plants’ MS traits [12], variation in the evaluated traits’ expression indicated satisfactory level of uniformity for paternal inbred line, considering that the STDEV values for all traits were below those for comparable L*c* line. On the other hand, STDEV values for certain evaluated traits observed in F1*exp* and its maternal inbred line were above those for comparable F*c* and L*c* varieties, although not exceeded the defined threshold value 1.26 for a sample of 40 plants in size [13], as was the case in this study, also indicating a satisfactory level of their uniformity.

It was reported that the degradation of measurement scale from scale to ordinal level significantly decreases environmental effects on the quantitative traits. The results obtained using such a biometric method have shown to be more reliable for genotypes comparison than the results based on mean values of the scale measurements over several years or locations [19]. Hence, according to the off-types approach, the maternal inbred exhibited more than two out of five levels of trait expression for the following traits: the anthocyanin coloration of tassel anthers, silks and brace roots as quantitative traits, and the type of grain as qualitative trait, respectively, which was even more pronounced in the experimental F1*exp* hybrid. It is well known that plant developmental stage may induce the variation in morphological traits’ expression [20]. As such, maize silk, usually light green in color at the initial phase, may become red, yellow, light brown or reddish-brown upon maturation [21]. Two consecutive ratings of trait expression marked as slight variations can be neglected since the evaluated traits are strongly dependent upon plant developmental stage. In addition, even if the part of the variation was attributed to the micro-environmental impact and the subjectivity of the examiner, high variation in several traits, often on different plants, as was the case in the present study, more likely indicated that the observed non-uniformity has a genetic background. This could be the consequence of the parental non-uniformity and/or out-cross pollination during hybrid seed production [2]. The higher level of heterogeneity observed in maternal line could be most likely attributed to the earlier generation of inbreeding, although pollen contamination and/or seed admixture during maintenance breeding could not be completely ruled out [22]. Since, as a rule, the states of expression for qualitative traits are not influenced by the environment [23], in this study, a part of the variation in type of grain trait could be attributed to the xenia effect because pollen effect could potentially cause the modification in the biochemical constituents of maize kernels [24].

### 4.2. Genetic Purity Estimation by Protein Markers

In this study, the conventional method of genetic purity assessment was conducted in the field, based on morphological traits. However, phenotypic uniformity assay could not provide information on the purity of specific genetic attributes that relate to the grain quality of examined maize genotypes.

Seed storage proteins have been used as biochemical markers to estimate genetic purity in many plant species [8,25,26,27]. Their advantages include high stability under any set of environmental conditions, inheritance in an additive way and genotype dependence regarding the presence and position of storage proteins’ bands identified by isoelectric focusing (IEF) [28]. A modification of IEF on polyacrylamide gel with a thickness of less than 0.15 mm is called ultrathin-layer isoelectric focusing (UTLIEF) and offers a faster, safer and cheaper technique for protein separation [29,30]. For this reason, UTLIEF method for variety identification and genetic purity testing was applied in this study.

It was reported that, in the process of seed genetic purity control, UTLIEF method enable the distinguishing between maternal and true F1 seeds, the presence of self-pollinated seeds in hybrid seed stocks and the contamination caused by unrelated lines’ pollination [31,32,33,34]. However, in the present study, UTLIEF separation of albumins and prolamins of individual seeds did not enable detection of a specific paternal marker band, making it impossible to determine potential selfing rate. Additionally, no genetic impurities in the tested F1*exp* hybrid were found. In chapter 8.9.3 of ISTA Rules [14], two different ampholyte pH ranges in gradient UTLIEF gels (composition of pH 2–4/4–6/5–8/4–9 and pH 5–8/2–11) are proposed. In this study, pH range 5–8/2–11 was the source of pH gradient in the gels, which might have led to lower possibility of detecting potential protein bands that would focus at the other overlapping pH regions (i.e., pH 2–4/4–6/5–8/4–9).

The conventional method of genetic purity assessment conducted in the field revealed off-types in F1*exp* and maternal line, which was opposite to UTLIEF results on genetic purity. Based on the results obtained, it can be suggested that the similar genetic makeup of parental components when seed storage proteins are concerned could lead to low potency of UTLIEF method in determining hybrid purity, which could have been the case with F1*exp* purity. The question often arises whether biochemical and molecular methods for determining purity need to reflect genetic differences related to traditional morphological traits. For practical purposes, the genetic approaches must optimally be able to identify seedlots that will express genetically based morphological or chemical differences that would be of concern to the farmer and industrial customers, even if those differences have no agronomic significance. In the case of maize, customers will not be satisfied with a crop that is genetically pure for a set of isozyme or DNA loci, but which expresses variability for plant height or kernel type that exceeds the normal bounds of experience or expectation. Because isozymes and genes affecting morphological traits are most usually coded by different and unlinked loci, a “clean” isozyme profile will not necessarily correlate with morphological homogeneity [35].

### 4.3. Genetic Purity Estimation by Molecular Markers

Assessment of genetic purity of parental inbred lines and parent-offspring test for the resulting F1 hybrids is an essential quality control function in maize hybrid breeding [22]. SSRs have proven to be particularly useful for providing breeders and geneticists with a tool to link phenotypic and genotypic variation, due to co-dominant inheritance pattern, high levels of polymorphism, multiallelic nature, reproducibility and the ability to detect polymorphism in closely related lines [36,37,38]. For these reasons, in the present study, the quality control genotyping, i.e., genetic purity estimation by SSR markers, was done to detect any potential contamination which could have happened during F1*exp* maize hybrid development and its parental inbred lines maintenance.

Out of 10 SSR primer pairs used, 2 SSRs (*phi*015 and *umc*1133), which showed the polymorphism between parental lines and confirmed their uniformity, also detected non-parental alleles in all F1*exp* plant samples, showing longer fragments than parental alleles; furthermore, this was in line with the reported occurrence of non-parental banding patterns in RIL progeny compared with the individual lines using simple-sequence repeat primers [39]. There are several factors which can lead to appearance of non-parental alleles.

Expansion in SSR length can occur through unequal crossover, leading to a profile pattern for progeny samples that differs from the parental lines. The use of tri- and tetra-nucleotide repeat motifs SSR markers in the present study, excludes, to a higher extent, the possibility that SSR regions might be affected by recombination due to reported high affinity of recombination enzymes towards dinucleotide repeat sequences [40]. However, mutation as a heritable change, distinct from recombination. The misalignment of DNA strands during the replication of repeated DNA sequences can lead to genetic rearrangements such as microsatellite instability, and if it occurs in the primer strand, base pairs will be added, resulting in a strand that is longer than the parental one [41]. Because dinucleotide motifs are highly prone to mutation, mutation also should not be considered as a potential causative source of non-parental bands’ occurrence in F1*exp* maize hybrid [42]. Although in this study there is no evidence of the marker position in the heterochromatin region, it could be possible that non-parental bands observed in F1*exp* resulted from chromosomal aberrations caused by rearrangement, due to the ability of the repetitive microsatellite DNA sequences to change their copy number is thought to promote random chromosomal rearrangements [43]. Moreover, because transposons are responsible for various chromosomal rearrangements and they participate in insertion mutagenesis [44,45], they may also play a role in the appearance of non-parental inheritance in F1*exp* maize hybrid [46].

However, a percentage of gene loci remains heterozygous despite inbreeding, and even a moderate advantage of heterozygotes over homozygotes can inhibit the process of obtaining homozygosity. In this study, only *umc*1545, which showed the polymorphism between parental lines and confirmed the uniformity of paternal inbred, detected non-specific bands in maternal plant samples recognized as off-types. Obtained results confirmed that a single co-dominant marker is sufficient to discern false hybrids in purity assessment [47], strongly recommending this SSR marker as a good candidate for genetic purity identification. The more pronounced occurrence of non-specific bands (i.e., off-types) in F1*exp* hybrid clearly confirmed that the purity level of the parental inbred lines determined the purity of the resulting F1*exp* hybrid, i.e., that the residual heterozygosity within parental inbred lines lead to appearance of non-specific bands in progeny [5,48,49]. Although, in this study, the applicability in genetic purity estimation was presented for only one pair of inbred-hybrid combination, this SSR marker has already been shown to be efficient in genetic purity testing of 15 inbreds as parental lines of MRIZP released hybrids.

Currently, most maize breeding programs consider S4 or later generation as a fixed inbred line for evaluation in hybrid combination. Pure or fixed are considered the inbreds in which the portion of heterozygous SSR loci does not exceed 5% [16]. In opposite, inbred lines with higher than 5% heterogeneous SSR loci are considered either not fixed (i.e., in the early generation of inbreeding) or likely to have been contaminated by pollen or seed of another source during seed regeneration, maintenance breeding and bulking [50]. It was reported that 21% of CIMMYT’s and 30% of IITA’s inbred lines showed heterogeneity values ranging from 12.5% to 31.5% [51]. In this study, paternal inbred exhibited 100% genetic purity according to SSR analysis. However, maternal inbred line had residual heterozygosity of 17.5%, which is significantly higher than the threshold of 5%. Lines with more than 15% residual heterozygosity are likely to have been contaminated with pollen from unrelated genetic materials and are required to be subjected to additional generations of inbreeding and to an extensive reselection for the original genotype [16].

Both the SSR and UPOV VS morphological markers showed homogeneity of paternal inbred; however, traditional morphological assay detected a higher percentage of genetic impurities of maternal inbred for 5% and of derived F1*exp* hybrid for 10%, respectively, compared to molecular marker-based assay. These findings confirmed that a strict correlation between molecular and morphological differences will only be possible if there is tight linkage between the molecular marker loci and the loci that form the genetic basis for expression of the morphological traits and if environmental factors do not significantly affect their expression [35].

## 5. Conclusions

Regardless of undertaking the clearly defined measures, potential mismatches can happen during maize hybrid development and parental inbred lines’ maintenance. To avoid this, pre-control test for varietal verification was applied, as a very important component of a seed multiplication and certification program, due to its ability to identify insufficiencies in varietal purity (i.e., shifts from trueness to type and in expression of varietal distinguishing characteristics) at an early stage, before causing high financial problems for all participants in the seed production chain. In this study, detected genetic impurities in one of the parental lines and derived F1*exp* hybrid by SSRs as environmentally independent genetic markers, indicated the importance of their use in pre-control seed quality testing, immediately after the harvest of seed production, and before the certification, packaging and sale of both F1 and parental lines maize seeds. Although, according to ISTA rules, sample sizes of greater than 100 may be required for precise varietal purity estimation, the identification of microsatellite marker *umc*1545 polymorphism and its ability to detect off-types in maternal line and resulting F1*exp* hybrid with 40 individuals in size are the most important results of this study. According to the analyses, this SSR marker could be recommended for quick and reliable maize genetic purity testing.

## Figures and Tables

**Table 1:** The list of observed morphological traits from the UPOV guidelines, developmental stage for the assessment (DSA), type of observation (TO) and range of notes by the descriptor (RND) [2,11].

No	List of Morphological Traits	DSA	TO	RND
3	Intensity of leaf green color	Inflorescence visible	VS	1–3
4	Undulation of leaf blade margin	Inflorescence visible	VS	1–3
5	Angle between blade and stem	Anthesis	VS	1–9
6	Curvature of leaf blade	Anthesis	VS	1–9
7	Degree of stem zig-zag	Anthesis	VS	1–3
9	Anth. col. at base of tassel glume	Anthesis	VS	1–9
10	Anth. col. of tassel glume exclude base	Anthesis	VS	1–9
11	Anth. col. of tassel anthers	Anthesis	VS	1–9
12	Angle btw. main axis and lateral tass. br.	Anthesis	VS	1–9
13	Curvature of lateral tass. branches	Anthesis	VS	1–9
14	Number of primary tassel branches	Anthesis to milk devel.	MS	no.
16	Anthocyanin coloration of silks	Anthesishafway	VS	1–9
17	Anthocyanin coloration of brace roots	Anthesis to milk devel.	VS	1–9
18	Density of tassel spikelets	Anthesis to watery ripe	VS	3–7
19	Anthocyanin coloration of sheath	Watery ripe to milk	VS	1–9
20	Anthocyanin coloration of internodes	Watery ripe to milk	VS	1–9
21	Length of main t. axis above lowest l. b.	Watery ripe to milk	MS	cm
22	Length of main t. axis above upper l. b.	Watery ripe to milk	MS	cm
23	Length of lateral branch	Watery ripe to milk	VS	1–9
24	Height of plant	Milk to dough develop.	MS	cm
25	Ratio Height of ear/ Height of plant	Milk to dough develop.	MS	cm
26	Width of blade	Milk to dough develop.	MS	cm
27	Length of ear peduncle	Milk to dough develop.	VS	1–9
28	Length of ear	After harvest	MS	cm
29	Diameter of ear in middle	After harvest	MS	cm
30	Shape of ear	After harvest	VS	1–3
31	Number of rows of grain	After harvest	MS	no
36	Type of grain	After harvest	VS	1–9
38	Color of top of grain	After harvest	VS	1–9
39	Color of dorsal side of grain	After harvest	VS	1–9
41	Anthocyanin coloration of glumes of cob	After harvest	VS	1–9

Abbreviations: No—order of traits in the UPOV descriptor; VS—Visual assessment of a number of individual plants or parts of plants; MS—Metric measurement of a number of individual plants or parts of plants.

**Table 2:** The list of SSR primers used for genetic purity assay of F1*exp* maize hybrid and its parental inbred lines.

SSR Markers	Bin	Repeat	Forward Sequence (5’-3’)	Reverse Sequence (5’-3’)
*phi*109275	1.03	AGCT	CGGTTCATGCTAGCTCTGC	GTTGTGGCTGTGGTGGTG
*phi*083	2.04	AGCT	CAAACATCAGCCAGAGACAAGGAC	ATTCATCGACGCGTCACAGTCTACT
*umc*1448	2.04	(GCT)5	ATCCTCTCATCTTTAGGTCCACCG	CATATACAGTCTCTTCTGGCTGCTCA
*phi*102228	3.06	AAGC	ATTCCGACGCAATCAACA	TTCATCTCCTCCAGGAGCCTT
*umc*1117	4.04	(TCGCA)4	AATTCTAGTCCTGGGTCGGAACTC	CGTGGCCGTGGAGTCTACTACT
*umc*1478	5.01	(GGAG)4	GAAGCTTCTCCTCTCGCGTCTC	CAGTCCCAGACCCTAGCTCAGTC
*umc*1133	6.01	ATAC	ATTCGATCTAGGGTTTGGGTTCAG	GATGCAGTAGCATGCTGGATGTAG
*umc*1545	7.00	(AAGA)4	GAAAACTGCATCAACAACAAGCTG	ATTGGTTGGTTCTTGCTTCCATTA
*phi*015	8.08	AAAC	GCAACGTACCGTACCTTTCCGA	ACGCTGCATTCAATTACCGGGAAG
*umc*1061	10.06	(TCG)6	AGCAGGAGTACCCATGAAAGTCC	TATCACAGCACGAAGCGATAGATG

**Table 3:** The uniformity assessment of the evaluated maize experimental hybrid and its parental inbred lines based on the STDEV approach for the metric traits.

No	Plant Trait	Mean Value					STDEV Value				
		F1*c*	F1*exp*	L*c*	F1♀	F1♂	F1*c*	F1*exp*	L*c*	F1♀	F1♂
14	NPTB	7.8	7.2	7.4	6.8	1.42	2.7	1.7	1.3	1.41	0.8
21	PHLTLB	206.2	190.7	161.6	175.7	92.0	18.9	18.5	13.8	10.1	13.0
22	PHHTLB	217.1	203.3	175.2	183.6	92.3	17.8	17.7	14.1	10.4	13.2
24	HP	242.1	229.0	196.5	206.3	114.9	18.1	17.8	14.3	11.2	13.6
25	HE	104.5	63.1	70.5	58.2	27.4	10.2	9.3	9.0	8.1	4.7
26	WB	9.6	9.7	9.15	7.4	6.6	1.6	1.7	0.8	0.5	0.7
28	LE	19.5	18.8	18.1	13.3	8.6	1.7	1.9	2.4	2.0	1.8
29	DE	4.5	4.2	3.3	3.6	2.9	0.3	0.2	0.3	0.3	0.2
31	NR	14.4	14.7	14.2	13.5	10.9	1.2	1.4	1.7	1.5	1.6

Abbreviations: No—number of characteristic in UPOV descriptor; NPTB—Number of primary tassel branches; PHLLB—Plant height to lowest tassel lateral branch; PHHLB—Plant height to highest tassel lateral branch; HP—Height of plant; HE—Height of ear; WB—Width of blade; LE—Length of ear; DE—Diameter of ear in middle; NR—Number of rows of grain; No—number of characteristic in UPOV descriptor; F1*exp*—experimental hybrid; F1*c*—comparable hybrid; F1♀—female parent; F1♂—male parent; L*c*—comparable inbred line.

**Table 4:** The uniformity assessment of the evaluated maize hybrid and its parental inbred lines based on the off-types approach for the visually assessed traits.

No	List of Morphological Traits	Note			Off-Type Note		
		F1*exp*	F1♀	F1♂	F1*exp*	F1♀	F1♂
3	ILGC	2	2	1			
4	ULBM	1	1	1			
5	ABLB	1	1	3			
6	CLB	3	1	1			
7	DSZ	1	1	1			
9	ACBG	1	1	1			
10	ACG	3	3	1	–	–	3-**1p**
11	ACA	5	5	1	1-**1p**; 7-**1p**	1-**2p**	–
12	ABMALB	5	3	1			
13	CLTB	3	5	1			
16	ACS	5-**16p**; 3-**20p**	1-**17p**; 3-**23p**	3	1-**3p**; 7-**1p**	–	–
17	ACBR	5-**31p**; 7-**8p**	5-**28p**; 7-**10p**	7(0*)	3-**1p**	3-**2p**	–
18	DTS	3	5	1	–	3-**1p**	–
19	ACSh	1	1	1			
20	ACIn	1	1	3-**17p**; 5-**23p**			
23	LLB	5	5	3	9-**1p**	–	–
27	LEP	5	5	1			
30	ShE	2	2	3			
36	TG	4-**13p**; 5-**22p**	5	1	3-**5p**	–	–
38	CTG	3-**26p**;4-**14p**	3	4	–	4-**4p**	–
39	CDSG	5	5	4	4-**1p**	–	–
41	ACGCb	1	1	1			
Σ Off type plants					**14p**	**9p**	**1p**

Abbreviations: No—number of characteristic in UPOV descriptor; F1*exp*—experimental hybrid; F1♀—female parent; F1♂—male parent; ILGC—Intensity of leaf green color; ULBM—Undulation of leaf blade margin; ABLB—Angle between leaf blade and stem; CLB—Curvature of leaf blade; DSZ—Degree of stem zig-zag; ACBG—Anthocyanin coloration at base of tassel glume; ACG—Anthocyanin coloration of tassel glume excluding base; ACA—Anthocyanin coloration of tassel anthers; ABMALB—Angle between main axis and lateral tassel branches; CLB—Curvature of lateral tassel branches; ACS—Anthocyanin coloration of silks; ACBR—Anthocyanin coloration of brace roots; DTS—Density of tassel spikelets; ACSh—Anthocyanin coloration of sheath; ACIn—Anthocyanin coloration of internodes; LLB—Length of lateral branch; LEP—Length of ear peduncle; ShE—Shape of ear; TG –Type of grain; CTG—Color of top of grain; CDSG—Color of dorsal side of grain; ACGC—Anthocyanin coloration of glumes of cob; OT—number of off-type plants for each characteristic; *—some plants have not developed brace roots. Bold text represents the number of plants. Red bold text stands for the number of off-type plants.

**Table 5:** The polymorphism detected by SSR markers.

Primer	F1♂	F1♀	F1*exp*
*phi*109275	*	*	*
*phi*083	*	*	*
*umc*1448	*	*	*
*phi*102228	*	*	non-parental bands in all
*umc*1117	*	*	non-parental bands
*umc*1478	*	*	non-parental bands
*umc*1133	uniform	uniform	non-parental bands in all
*umc*1545	uniform	non-specific band	F1♀ non-specific band
*phi*015	uniform	uniform	non-parental band in all
*umc*1061	*	*	non-parental bands

*—no polymorphism detected between the parental lines

**Table 6:** The SSR markers and their allele size (bp).

Primer	F1♂	F1♀	F1*exp*	F1*exp* Non-Specific/Non-Parental Band	F1♀ Non-Specific Band
*phi*109275	130 bp	130 bp	130 bp	–	–
*phi*083	202 bp	202 bp	202 bp	–	–
*umc*1448	154 bp	154 bp	154 bp	–	–
*phi*102228	138 bp	138 bp	138 bp	155/185 bp	–
*umc*1117	120/125 bp	120/125 bp	120/125 bp	202/223 bp	–
*umc*1478	135/140 bp	135 bp	135 bp	195/245 bp	––
*umc*1133	102 bp	91 bp	91/102 bp	165/184 bp	–
*umc*1545	85 bp	77 bp	77/85 bp	92 bp	92 bp
*phi*015	135 bp	127 bp	127/135 bp	187 bp	–
*umc* 1061	106 bp	106 bp	106 bp	125/137 bp	–

## Data Availability

The data presented in this study are available in the article.
